# Targeting mu opioid receptors to modulate gastrointestinal function: what have we learnt so far from the studies in functional bowel disorders?

**DOI:** 10.12688/f1000research.15974.1

**Published:** 2019-03-05

**Authors:** Maura Corsetti, Jasper Pannemans, Peter Whorwell

**Affiliations:** 1 NIHR Nottingham Biomedical Research Centre (BRC), Nottingham University Hospitals NHS Trust and the University of Nottingham, Nottingham, UK; 2 Nottingham Digestive Diseases Centre, School of Medicine, University of Nottingham, Nottingham, UK; 3 Catholic University of Leuven, KU Leuven, Translational Research Center for Gastrointestinal Disorders (TARGID), Leuven, Belgium; 4 Centre for Gastrointestinal Sciences, University of Manchester, Manchester, UK

**Keywords:** opioids, mu opioid receptors, PAMORA, eluxadoline

## Abstract

Opioids have recently received much attention because of the epidemic in their use in some countries such as the USA and the UK. Concerns have been raised about the possibility that they can increase mortality in patients when used on a long-term basis. Moreover, they are known to induce paradoxical hyperalgesia as well as alterations of gut function. The analgesic properties of opioids are mediated by receptors located in the brain, but as opioid receptors are also expressed in the gastrointestinal tract, new drugs acting on these receptors have recently been developed to treat two functional disorders, namely irritable bowel syndrome with diarrhoea and opioid-induced constipation. The aim of this article is to highlight some interesting observations resulting from the development of these drugs in the field of functional gastrointestinal disorders.

## Introduction

Opioids have recently received much attention because of the epidemic in their use in some countries such as the USA and the UK. They are the most commonly prescribed class of drugs to treat chronic pain, particularly non-cancer pain, in the USA ^1^. Concerns about the possible increased mortality associated with the use of these medications and the paradoxical hyperalgesia they can induce have raised questions about their role in the management of non-cancer pain, particularly when used on a long-term basis ^2^. The analgesic properties of opioids are mediated by receptors located in the brain, but as opioid receptors are also expressed in the gastrointestinal (GI) tract, new drugs acting on these receptors have recently been developed to treat irritable bowel syndrome with diarrhoea (IBS-D) and opioid-induced constipation (OIC). In a recent article two of the co-authors of the present article reviewed the data concerning these new drugs ^3^. The aim of this review is to highlight some interesting observations resulting from the development of these drugs in the field of functional gastrointestinal disorders.

## What do we know about the effect of opioids on gastrointestinal function?

µ, κ, δ, and opioid receptor-like-1 (ORL-4) ^4^ receptors are G protein–coupled receptors found in the central nervous system. In the GI tract, at the site of µ, κ and δ receptors ^3^, opioids are able to bind to opioid receptors.

Most of the GI effects seem to be mediated by the high number of µ-receptors expressed on neurons of the enteric nervous system ^4, 5^. In general, opioids have been demonstrated to reduce gut transit by stimulating non-propulsive contractions and increasing the tone of gut sphincters ^5^. Opioids have been associated with increased lower oesophageal sphincter (LOS) resting pressure, decreased LOS relaxation, and reduced number of transient LOS relaxations ^6^. Opioids have been demonstrated to delay both gastric emptying and small bowel transit as well as to impair bile duct flow, possibly as a result of increased frequency in sphincter of Oddi contractions ^5^. Opioids cause decreased colonic propulsion through inhibition of excitatory neural pathways as well as inhibition of inhibitory neural pathways leading to non-propulsive colonic motility, eventually resulting in constipation. In addition, they lead to concomitant inhibition of electrolyte and water secretion and increased absorption of water and electrolytes ^5^. At the distal end of the colon, opioids have also been shown to increase the rectal distension threshold for first perception and for inducing recto-anal inhibitory reflex ^6^. The effect of opioid receptor activation on gut motility has been attributed to the pre-synaptic inhibition of the release of excitatory transmitters from neurons governing smooth muscle contractions ^6^. However, studies on animal and human colons have demonstrated that inhibition of inhibitory neuromuscular transmission may also play a role ^6^. Interestingly, although most of the effects induced by opioids exhibit tolerance, this does not seem to be the case for GI consequences ^6^. Recent studies have suggested that this could be due to differences or alterations of the receptor signalling mechanisms in enteric as compared with central neurons during prolonged opioid use ^6^.

## What have we learnt from the use of mu opioid receptor agonists in the treatment of diarrhoea in irritable bowel syndrome?

IBS is a functional GI disorder characterised by the presence of recurrent abdominal pain associated with abnormal stool frequency and form ^7^. In addition, the majority of patients complain of abdominal bloating, which often is accompanied by visible distension of the abdomen. The prevalence of IBS is about 10% in Western countries, and owing to its chronic relapsing course, it represents a major cost to healthcare resources as these patients use healthcare services more than the general population ^7, 8^. Patients with IBS-D represent about one third of the IBS population and, compared with patients who have IBS with constipation, experience more pain attacks, abdominal pain that is more related to bowel movements, more urgency, a less stable bowel habit, and less bloating ^7, 8^.

The treatment of IBS is normally based on reassurance and education coupled with dietary and lifestyle advice ^8^. The traditional pharmacological treatment of IBS-D includes the combination of drugs acting on pain (antispasmodics) and diarrhoea (loperamide), and antidepressants are used for those not responding to initial therapy. Recently, both the US Food and Drug Administration (FDA) and the European Medicines Agency (EMA) have approved eluxadoline, a mixed µ- and κ-opioid receptor agonist and δ-opioid receptor antagonist, for the treatment of IBS-D.

Eluxadoline is not the first µ-opioid receptor agonist used in IBS-D. Loperamide, an opiate analogue of the piperidine class with low bioavailability, has been used to treat acute and chronic diarrhoea for over three decades. Owing to its µ-opioid receptor agonist activity, loperamide has limited penetration of the blood–brain barrier and inhibits GI peristalsis and secretion ^9^. In total, four trials have evaluated the efficacy of loperamide in IBS patients with diarrhoea or mixed stool patterns ^10^. Loperamide showed an effect on stool consistency, urgency and bowel frequency, associated with delay in both small bowel and whole gut transit time. Although some trials have demonstrated pain reduction, systematic reviews do not advocate the use of loperamide in IBS-D owing to insufficient evidence ^10^. Despite this, in clinical practice, loperamide is the most commonly used drug for IBS-D. Side effects of loperamide include nausea, vomiting, constipation, dry mouth, dizziness, and abdominal discomfort ^10^. Recent warnings have reported that abuse or misuse of loperamide at very high doses can lead to cardiac adverse events (AEs) ^11^.

Eluxadoline is a phenylimidazole with mixed opioid receptor activity, acting as a µ-opioid receptor agonist with a binding affinity (Ki) of 1.8 nmol/L for the human receptor, a δ-opioid receptor antagonist with a Ki of 430 nmol/L for the human receptor, and a κ-opioid receptor agonist with an as-yet-unknown Ki for the human receptor ^8^. The exact mechanism of action of eluxadoline is unclear. However, studies have found that the antagonism of the δ-opioid receptor may enhance the analgesic effect of morphine, preventing the induction of tolerance, suggesting the presence of an interaction between the two receptors ^8^.

Two multicentre, double-blind, placebo-controlled, phase 3 studies, conducted in 2428 patients with IBS-D, have been reported ^12^. These were 26-week studies followed by a 26-week follow-up period and a 2-week post-treatment follow-up period in one trial and by a 4-week withdrawal period in the other trial. Patients were considered to be “responders” when they experienced at least a 30% reduction from their baseline score of worst abdominal pain in at least 50% of the days and a stool consistency score of less than 5 on the Bristol stool scale over 12 and 26 weeks, respectively, according to the FDA and EMA endpoints.

The percentage of patients, defined as responders to twice-daily 75 mg and 100 mg eluxadoline, according to the composite FDA endpoints, was higher as compared with placebo (23.9% and 25.1% respectively versus 17.1%, *p* = 0.004; 28.9% and 28.9% respectively versus 16.2%, *p* <0.001). Furthermore, the percentage of patients, defined as responders according to the composite EMA endpoints, was significantly higher as compared with placebo, but in this case only for the 100-mg dose (29.3% versus 19.0%, *p* <0.001 and 32.7% versus 20.2%, *p* <0.001). The number needed to treat for eluxadoline is 8 ^13^.

Both doses showed superiority to placebo for stool consistency, frequency, urgency, adequate relief of IBS symptoms, global symptom scores, and scores on IBS-quality of life (IBS-QOL) questionnaires. However, when only the percentage of patients who reported an improvement of at least 30% in their worst abdominal pain was considered, this was not significantly higher than placebo.

A post-hoc analysis, focusing on loperamide use before and during the trials, revealed that about 36% of the patients reported prior use of loperamide and that 59% to 67% of these had inadequate IBS-D symptom control on loperamide ^14^. Patients who reported adequate symptom control with earlier use of loperamide were more likely to be composite responders to eluxadoline compared with placebo (44.3% versus 26.7% respectively, *P* <0.01). However, when daily rescue loperamide use was imputed as a non-response day, the composite responder rate was still higher in patients receiving eluxadoline as compared with placebo over weeks 1 to 12 and weeks 1 to 26 for both dosages.

The most common AEs when taking eluxadoline were nausea, constipation, and abdominal pain ^12^. However, a more serious side effect of pancreatitis was reported in some patients participating in the pivotal trials.

In a recent editorial by Chedid *et al*. ^15^, reviewing 1666 patients who received eluxadoline in the phase 3 trials, five developed acute pancreatitis: two on 75 mg and three on 100 mg. Furthermore, there have been 120 reports of pancreatitis or death in patients receiving eluxadoline made to the FDA via the Federal Adverse Events Reporting System (FAERS), a publicly accessible reporting system. As a result, the FDA released a Drug Safety Communication warning detailing the heightened risk of pancreatitis with the use of eluxadoline in patients with IBS-D who had undergone prior cholecystectomy. The FDA declared previous cholecystectomy a contraindication for the use of eluxadoline in line with a previous recommendation by the EMA. Some incidents occurred after only one or a few doses of the drug. Acute pancreatitis has also been documented in cases where there was no prior cholecystectomy. These cases of pancreatitis following the use of eluxadoline were assumed to be the result of sphincter of Oddi spasm. The definition of pancreatitis was based on the Atlanta criteria, a universally applicable classification system for the diagnosis of acute pancreatitis based on clinical criteria and radiological criteria as well as clinical judgement. The problem with the FAERS is that it is a voluntary reporting system, making it difficult to calculate the true incidence of pancreatitis in patients taking eluxadoline, especially as this problem can be seen with other drugs used for controlling diarrhoea in IBS, such as loperamide and other opiates. Consequently, it would be useful to have a study evaluating the incidence of pancreatitis in patients taking loperamide and possibly other opiates, as not many clinicians are aware of this potential side effect. In order to possibly mitigate this problem with eluxadoline, a formulation designed to specifically target the colon is in development ^16^. Alcoholism, alcohol abuse, alcohol addiction, and chronic or acute excessive alcohol use are other important contraindications because these patients already have an increased risk of acute pancreatitis.

The problem of side effects and in particular serious side effects, such as those reported for eluxadoline or in the past with the 5-HT3 antagonist alosetron (ischemic colitis), is that IBS is usually considered a completely benign condition. However, it has been consistently shown that IBS can have a profound impact on the QOL of patients with IBS. Indeed, 13.5% of patients would be willing to accept at least a 1-in-1000 chance of death and 10.1% would accept at least a 1-in-1000 risk of serious or permanent side effects from a medication to achieve perfect health ^17^. Patients with the most severe IBS were the most likely to accept risk. In secondary and tertiary care, there is a relationship between IBS and significant suicide risk, which is not associated with any concurrent depression ^18^.

Current evidence shows that eluxadoline is less likely to be abused than other schedule II µ-opioid receptor agonists among recreational opioid users. The use of supratherapeutic doses of oral or intranasal eluxadoline led to results not comparable to those observed with the use of the µ-agonist oxycodone (positive control), although some small but significant differences from placebo were observed for some outcome measures. However, the therapeutic dose of eluxadoline was similar to placebo following oral administration and intranasal eluxadoline was generally associated with significant dislike versus placebo and oxycodone ^19^.

## What have we learnt from the use of mu opioid receptor antagonists in the treatment of opioid-induced constipation?

As reported before, opioids are the most commonly prescribed class of drugs to treat chronic pain, particularly non-cancer pain. Based on a systematic review that included 15 randomised placebo-controlled trials ^20^, the prevalence of OIC in patients taking opioids for chronic non-cancer pain is 41%. This disorder was recently classified as part of the functional bowel disorders by the Rome Foundation and defined as “a change, when initiating opioid therapy, from baseline bowel habits and defecation patterns, that is characterised by any of the following: reduced bowel frequency; development or worsening of straining; a sense of incomplete evacuation; or a patient’s perception of distress related to bowel habits” ^7^. The occasional patient may also develop faecal impaction with overflow incontinence, whereas others may report symptoms compatible with overlapping opioid-induced bowel disorders (for example, reflux nausea and bloating) ^7^.

Although this disorder does not directly lead to an increase in mortality, it results in a reduction in QOL. In a recent cross-sectional analysis of an ongoing longitudinal study conducted in the USA, Canada, Germany and the UK on patients with self-reported OIC on daily opioid therapy for at least 4 weeks for non-cancer pain, it was reported that constipation symptoms limit the work productivity and overall health-related QOL of these patients ^21^. In a recent retrospective analysis in patients with non-cancer pain in the USA, it was demonstrated that among opioid users those with OIC generate significantly higher total healthcare costs as compared with those without ^22^.

OIC is normally managed with treatments similar to those applied in functional constipation, including fibre, stimulant and osmotic laxatives, lubiprostone, linaclotide and prucalopride. However, laxatives have not been found to be better than placebo and the others do not target the mechanism of OIC ^6^. Therefore, new drugs acting on µ-opioid receptors, including naloxegol, methylnaltrexone and naldemedine, have recently been developed to treat OIC. They are all peripherally acting µ-opioid receptor antagonists (PAMORA) and an oral formulation approved by the FDA and the EMA for this condition is available.

### Naloxegol

Naloxegol is a PAMORA derived from the µ-opioid receptor antagonist naloxone and is the first oral medication approved by both the FDA and the EMA for the treatment of OIC in patients with non-cancer pain. Preclinical investigations showed the ability of naloxegol to accelerate GI transit time in morphine-induced constipation without affecting the analgesic effects of morphine ^23^. Owing to PEGylation, attachment of polyethylene glycol causing P-glycoprotein transporter-substrate properties, the ability of naloxegol to cross the blood–brain barrier is limited ^23^.

Two randomised, double-blind, 12-week, phase 3 trials were conducted in patients with OIC and non-cancer pain to assess the effect of naloxegol 25 mg and 12.5 mg as compared with placebo ^24, 25^. According to the FDA and EMA endpoints, a responder was defined as a patient experiencing at least three spontaneous bowel movements (SBMs) weekly, with an at least one-SBM increase over baseline in at least 9 out of 12 weeks and at least 3 out of 4 final weeks. In the KODIAC 04 study, both doses achieved a higher response rate (RR) compared with placebo (25 mg: RR 1.51, 95% confidence interval (CI) 1.17–1.95, *p* = 0.001 and 12.5 mg: RR 1.38, 95% CI 1.06–1.80, *p* = 0.02). In KODIAC 05, only the 25-mg dose achieved a significant difference compared with placebo (25 mg: RR 1.35, 95% CI 1.05–1.74, *p* = 0.02 and 12.5 mg: RR 1.19, 95% CI 0.91–1.55, *p* = 0.20). In the laxative-inadequate response (LIR) subpopulation (defined as patients “who took laxatives in one or more laxative classes for a minimum of 4 days within 2 weeks before screening and had ratings of moderate, severe, or very severe on one or more of the four stool-symptom domains in the baseline laxative-response questionnaire” ^24^), which made up 53.9% of the total population, the 25-mg treatment group achieved a greater RR compared with placebo (KODIAC 04: RR 1.69 95% CI 1.21–2.37, *p* = 0.002 and KODIAC 05: RR 1.49, 95% CI 1.08–2.06, *p* = 0.01) ^24^. In addition, greater improvements were found with 25 mg naloxegol for straining, stool consistency, and frequency of days with SBM in both trials. Naloxegol was generally safe and well tolerated at a dose of 25 mg, and the most frequent AEs were GI-related, such as diarrhoea, abdominal pain and vomiting ^26, 27^. QOL was not measured in these trials.

### Methylnaltrexone

N-methylnaltrexone bromide is a quaternary derivative of naloxone PAMORA. Naloxone is effective in antagonising the inhibitory responses of morphine on smooth muscle and accelerating GI transit time ^28– 32^. The quaternary functional unit decreases lipid solubility, resulting in blood–brain barrier passage restriction ^28^. Methylnaltrexone is available as both subcutaneous and oral formulation. In healthy subjects, oral methylnaltrexone significantly attenuated or completely prevented morphine-induced delay in oro-cecal transit time, depending on the dose.

A previous multicentre, double-blind, randomised controlled phase 3 trial, including 460 patients with non-cancer OIC, was conducted to compare the efficacy of subcutaneous methylnaltrexone 12 mg once daily (QD) or every other day and placebo over 4 weeks ^32^. The co-primary efficacy endpoints were the proportion of patients having a rescue-free bowel movement (RFBM or bowel movement without previous assumption of rescue medication) within 4 hours of the first dose and the percentage of active injections per patient resulting in an RFBM within 4 hours. A greater percentage of patients who received methylnaltrexone QD or alternate-day dosing as compared with placebo were able to achieve an RFBM within 4 hours of the first dose (34.2% versus 9.9%, *p* <0.001). In addition, 28.9% of methylnaltrexone QD and 30.2% of methylnaltrexone alternate-day dosing resulted in RFBMs within 4 hours versus 9.4% QD and 9.3% alternate-day placebo injections (both *p* <0.001). Most common AEs were abdominal pain, nausea, diarrhoea, hyperhidrosis and vomiting. It could be argued that having an RFBM within 4 hours of the first dose is not of clinical relevance in a chronic condition, although this study also showed an improvement in QOL. At the end of the double-blind period (day 28), the methylnaltrexone QD group showed a significantly greater mean improvement of 0.74 (33%) from baseline in Patient Assessment of Constipation-QOL total scores compared with an improvement of 0.39 (18%) in patients receiving placebo ( *p* <0.001). At day 28, the patients receiving methylnaltrexone every other day showed a mean improvement of 0.59 (27%) from baseline, which was significantly greater than that seen in the placebo group ( *p* = 0.014) ^33^.

Because of the reported risk of perforation in studies evaluating the effect of methylnaltrexone in patients with cancer, the medication is not recommended to patients with OIC when it is associated with conditions of the GI tract potentially increasing this risk (that is, inflammation) ^34^.

Recently, a multicentre, double-blind, randomised controlled phase 3 trial, including 804 patients with non-cancer OIC, was conducted to compare the efficacy of oral methylnaltrexone (150, 300 or 450 mg) or placebo QD for 4 weeks followed by as-needed dosing for 8 weeks ^35^. Patients who had at least three RFBMs per week, with an increase of at least one RFBM per week from baseline for at least 3 out of 4 weeks during the QD period, were responders. The percentage of responders (49.3% for 300 mg and 51.5% for 450 mg versus 38.3% with placebo, all *p* <0.03) and change from baseline in mean number of weekly RFBMs (difference versus placebo, 0.5 for 300 mg and 0.5 for 450 mg, all *p* <0.03) were significantly greater with methylnaltrexone 300 and 450 mg/day versus placebo during the QD period. AEs were mostly GI-related; abdominal pain, nausea and diarrhoea were the most frequently reported. In 2016, the oral tablet received FDA approval for use in patients with OIC. QOL was not measured in this trial.

### Naldemedine

Naldemedine (S-297995) is a PAROMA that was approved by the FDA in 2017 for the treatment of OIC. Its structure is similar to that of naltrexone; however, it possesses an extra side chain, which increases its molecular weight and polar surface area, thereby limiting its ability to cross the blood–brain barrier ^36, 37^.

Two, 12-week, multicentre, phase 3 trials have been conducted to evaluate the efficacy of oral naldemedine 0.2 mg or matching placebo QD for 12 weeks in patients with chronic non-cancer pain ^35^. In these studies, the same FDA and EMA endpoints employed in the naloxegol trials reported above were used ^21^. In COMPOSE I and II, 547 and 553 patients, respectively, were assigned to naldemedine or placebo. In both trials, the percentage of responders was significantly greater in the naldemedine group compared with the placebo group (47.6% versus 34.6%, *p* = 0.002 in COMPOSE I and 52.5% versus 33.6%, *p* <0.001 in COMPOSE II). The drug was well tolerated, although more GI AEs such as diarrhoea and abdominal pain were observed.

The long-term safety of naldemedine was subsequently evaluated in 1246 patients with OIC and chronic non-cancer pain in a 52-week, randomised, double-blind, phase 3 study ^38^. The proportion of patients experiencing a treatment-related AE was similar between the two groups (68.4% with naldemedine versus 72.1% with placebo). Bowel movement frequency and overall constipation-related symptoms and QOL were improved with the use of naldemedine and these benefits did not affect opioid-mediated analgesia or necessitate opioid withdrawal.

## Conclusions

IBS-D and OIC are two common disorders, which are challenging to treat and have a significant negative impact on the QOL of patients. The prevalence of OIC is likely to increase as a result of the ‘opioid epidemic’ ^1^ and therefore there is a strong need for better management strategies.

Eluxadoline is a welcome addition to the therapeutic options for IBS-D as there are currently very few pharmacological alternatives. Currently, if loperamide is ineffective, ondansetron has been shown to be a good alternative ^10^ but if that fails, codeine is the only other option but it carries the risk of dependency. In this situation, in a patient who is desperate for some improvement, eluxadoline should be considered and is probably relatively safe as long as the exclusion criteria are strictly adhered to and patients are fully informed about the possible side effects. The authors are accumulating real-world clinical experience of eluxadoline in tertiary-care patients refractory to all other medications and finding it very effective in a significant proportion of individuals with this challenging form of IBS.

Obviously, the best treatment for OIC would be to stop the use of opioids as a long-term therapy, especially as there is accumulating evidence that opioids are ineffective in controlling non-cancer chronic pain syndromes. However, while trying to reduce this overuse, medications such as naloxegol, methylnaltrexone and naldemedine are welcome tools in helping to manage this problem, especially as they are relatively free of side effects. There have been no direct comparisons of these PAMORAs and therefore the choice of which one to use is likely to be based on what is currently available on the market in a particular country.

In light of the data accumulating in recent years on the use of opioids and µ-opioid receptor agonists and antagonists to modulate the GI function, some lessons can be learned. Opioid receptors have a significant impact on the function of the entire gut as demonstrated by preclinical studies which explain the side effects observed in clinical practice. It is clear from reviewing the activity of the drugs developed so far that worthwhile therapeutic effects can be achieved but, in some instances, at the expense of some AEs. Consequently, modulating opioid receptors deserves further study, especially as, after many years of little progress, new techniques of assessing the physiology of the GI system are becoming available.

## Abbreviations

AE, adverse event; EMA, European Medicines Agency; FAERS, Federal Adverse Events Reporting System; FDA, US Food and Drug Administration; GI, gastrointestinal; IBS, Irritable bowel syndrome; IBS-D, irritable bowel syndrome with diarrhoea; LOS, lower oesophageal sphincter; OIC, opioid-induced constipation; PAMORA, peripherally acting µ-opioid receptor antagonists; QD, once daily; QOL, quality of life; RFBM, rescue-free bowel movement; RR, response rate; SBM, spontaneous bowel movement
